# Efficacy and safety of omalizumab therapy in urticaria vasculitis

**DOI:** 10.3389/falgy.2022.952079

**Published:** 2022-07-22

**Authors:** Fiorella Petrelli, Daiana Giannini, Silvia Bilia, Isabella Del Corso, Valeria Rocchi, Paola Migliorini, Ilaria Puxeddu

**Affiliations:** Immunoallergology Unit, Department of Clinical and Experimental Medicine, Pisa University, Pisa, Italy

**Keywords:** urticarial vasculitis, omalizumab, anti IgE, therapy, biological agent

## Abstract

Urticarial vasculitis (UV) is a small-vessel leukocytoclastic vasculitis characterized by different clinical manifestations ranging from long-lasting urticarial lesions to severe and potentially life-threatening multi-organ involvement. Omalizumab (OMA), anti-IgE recombinant humanized IgG1 monoclonal antibody, has been successfully used to treat few cases of severe and/or refractory UV. In this study we report our experience on 6 patients with refractory normocomplementemic UV successfully treated with anti-IgE therapy (OMA), suggesting that this biological therapy may be a safe and effective therapeutic option in UV.

## Introduction

Urticarial vasculitis (UV) is a rare immune-complex mediated small-vessel leukocytoclastic vasculitis, characterized by long-lasting urticarial lesions, persisting more than 24 h. Clinical manifestations may range from itchy and/or burning skin lesions to severe and potentially life-threatening multi-organ involvement, having a substantial impact on patients' life expectancy and quality of life. On the basis of complement levels, UV is classified into two different forms: normocomplementemic (NUV) and hypocomplementemic (HUV). Moreover, the latter form is mainly associated with multi-organ involvement and with the presence of anti-C1q antibodies in more than half of the patients (1). Although most of the forms of UV are defined as idiopathic as the cause of the disease is not identified, some of them can be also associated with autoimmune and/or infectious and/or malignant diseases and can resolve following treatment of the underlying condition. Several drugs or vaccines, including recently those for COVID-19, have been identified as potential triggers for UV (1–3). The therapeutic approach to this disease is currently challenging due to the lack of large randomized controlled trials and approved therapies. Corticosteroids are among the most effective drugs for the treatment of both skin and extracutaneous manifestations, although their long-term administration may lead to serious dose-dependent side effects. In such cases immunosuppressive or immunomodulatory therapies such as azathioprine, cyclophosphamide or cyclosporine may improve disease control and reduce the dosage of corticosteroids. However, side effects, especially in the case of prolonged administration, and/or lack of efficacy often require discontinuation of the treatment (2, 4).

Thus, due to the evident limitations of the current treatment of UV, it is important to identify novel effective and safe therapeutic approaches for patients with diagnosis of UV, able to improve their quality of life and life expectancy. Several biological agents such as etanercept, infliximab, canakinumab, anakinra, rituximab, tocilizumab have been proposed as alternative therapeutic options in severe and/or treatment-refractory UV patients, even though the evidences on their efficacy and safety in the treatment of this condition are currently limited to a few case reports (2, 4).

Omalizumab (OMA) is a recombinant humanized IgG_1_ monoclonal antibody directed to IgE-specific epitopes within the C3 (FcεRI binding) region of circulating IgE, that reduces IgE/FcεRI binding on mast cells and basophils with consequent down-regulation of cell activation. After being approved in the USA and Europe for the treatment of antihistamine refractory chronic spontaneous urticaria (CSU), severe allergic asthma and severe chronic rhinosinusitis with nasal polyps, its efficacy and safety has been widely demonstrated in real-life experience. Based on the markedly positive impact of OMA in CSU, the use of this drug has been reported in some cases of severe and/or refractory UV either idiopathic or associated with autoimmune diseases (2, 4, 5).

In the Immuno-allergology Unit of Azienda Ospedaliero-Universitaria Pisana (AOUP) 6 patients with diagnosis of severe and/or refractory UV (F/M 5:1) with mean age of 62 years (range 43–74) were treated with OMA at dosage of 300 mg every 4 weeks, according to our experience in CSU (6). Written informed consent for off-label therapy was obtained from all the patients (University Hospital internal protocol, AOUP). Demographic and clinical characteristics were collected, including co-morbidities and current and previous pharmacological treatments for UV. According to the improvement in skin (long-lasting wheels, itch, burning, pain) and systemic (arthralgia, asthenia, abdominal pain, fever and lymphadenopathy) symptoms in response to OMA treatment, the patients were classified as responders, partially responders or not responders and any adverse side effects were recorded. The levels of IgE in the sera of UV patients were also measured before staring OMA treatment.

As reported in Table 1, in all the 6 patients evaluated the results of the immunohystochemistry analysis of skin biopsies were consistent with the diagnosis of UV, mainly revealing a lymphocytic/mononuclear infiltrate. Four out of the 6 patients reported systemic symptoms, including arthralgia, asthenia, abdominal pain, fever and lymphadenopathy, without ocular, renal, or cardiovascular involvement. According to the levels of complement components (C3 and C4), all UV evaluated were classified as NUV and none of them had anti-C1q antibodies. All of the patients had co-morbidities such as infectious diseases and/or malignancies and/or autoimmune diseases, however their UV were un-responsive to the pharmacological treatment for the underlying diseases. Before OMA treatment, all the patients had previously received oral corticosteroids, 5 of them also immunomodulatory and/or immunosuppressive drugs (see Table 1). Only one patient underwent previous biological therapies with anakinra and canakinumab due to severe and refractory disease. Reliever or daily administration of second generation H_1_-antihistamines were maintained in all the patients during the treatment with OMA. In 4 out of the 6 patients evaluated, a complete resolution of skin symptoms was achieved since the 1st OMA injection. As reported in Table 1, one patient was a partial responder (according to the improvement of skin symptoms) and this was observed after the 3rd injection. In most of our cohort, the improvement of both skin and systemic symptoms was observed since the 1st injection. Among the six patients evaluated, only one did not achieve any improvement in skin symptoms before the 4th OMA injection. She partially improved her long-lasting skin lesions from the 4th to the 6th injection and she was not responder anymore from the 7th injection. According to the clinical history of this patient, the discontinuation of OMA treatment was decided after the 10th injection.

**Table 1:** Demographic and clinical characteristics of patients with normocomplementemic UV.

**Pt**	**Sex**	**Age, yrs**	**Comorbidities**	**UV onset, yrs**	**UV duration, yrs**	**Systemic symptoms**	**Baseline IgE levels (U/ml)**	**Skin lesional biopsies**	**Previous therapies**	**OMA 300 mg/4 wks, injections (n**°**)**	**Response after (wks)**	**Skin symptoms improvement (after wks)**	**Systemic symptoms improvement (after wks)**	**SE**
1	F	43	HP infection, Hashimoto thyroiditis, IGT, liver disease	7	31	Yes	461	Lympho-monocytic	CS, CsA, AZA	10	16	Partial improvement (16), but no improvement (25)	No improvement	None
2	F	48	HP infection, Hashimoto thyroiditis	27	20	Yes	138	Lympho-monocytic	CS, HCQ	7	4	Complete resolution (4)	Complete resolution (4)	None
3	F	67	GERD, ACD, liver disease, osteopenia	53	10	Yes	177	Lymphocytic	CS, HCQ, CsA, LEF, CLH, AZA, MMF, MTX, anakinra, canakinumab	38	4	Complete resolution (4)	Partial improvement (4)	Headache
4	F	72	Asthma, DM2, AHT, liver disease	65	4	Yes	161	Granulocytic	CS, CsA, MTX, HCQ, MMF, DP	22	4	Complete resolution (4)	Complete resolution (4)	None
5	M	74	Latent TBC, MGUS, neoplatic disease, COPD, AHT, BPH	68	3	No	42	Lymphocytic/ granulocytic	CS	11	12	Partial improvement (12)	–	None
6	F	66	Neoplastic diseases	62	0.6	No	358	Granulocytic	CS, CLH	4	4	Complete resolution (4)	-	Maculo-Papular rash

*Pt, patient; F, female; M, male; yrs, years; HP, Helicobacter pylori; IGT, impaired glucose tolerance; GERD, gastroesophageal reflux disease; ACD, allergic contact dermatitis; DM2, diabetes mellitus type 2; AHT, arterial hypertension; TBC, tuberculosis; MGUS, monoclonal gammopathy of undetermined significance; COPD, chronic obstructive pulmonary disease; BPH, benign prostatic hyperplasia; UV, Urticarial vasculitis; C3, complement C3; C4, complement C4; CS, corticosteroids; CsA, cyclosporine; AZA, azathioprine; HCQ, hydroxicloroquine; LEF, leflunomide; CLH, colchicines; MMF, mycophenolate mofetil; MTX, methotrexate; DP, dapsone; OMA, Omalizumab; wks, weeks; no, number; SE, side effects*.

During the OMA treatment some common mild/moderate side effects such as headache were reported and in one case a maculo-papular rash occurred after the 4th injection.

In our experience, OMA, used at dosage of 300 mg every 4 weeks, proved to be a safe and effective therapeutic option in severe and/or refractory UV, for which no drugs are currently approved. Thus, the possibility to treat severe and/or refractory UV with manageable drugs such as OMA, that improves the quality of life of these patients, is an important goal for physicians managing UV patients. Few reports have already described the efficacy of OMA in treatment of different forms UV. However, the dosage of OMA and the interval between injections are not concordant among these clinical reports. This is probably due to the heterogenicity of the patients in terms of co-morbidities and/or clinical manifestations and/or laboratory data.

Even though the precise mechanisms of action of this biological agent are still to be clarified, we can postulate that its efficacy in UV treatment is probably due to its ability to reduce circulating IgE, and their binding to membrane of mast cells and basophils, that finally leads to downmodulation of cellular activation and/or inflammatory cells chemotaxis and/or immune complex formation (7).

A limit of our study is that it was a single arm open-label designed and further designed randomized double blind placebo control clinical studies are required. We have to take into account that in further studies large sample groups with different clinical phenotypes of UV should be included. On the whole our report suggests that OMA might be a promising option in the treatment of UV. The major improvement observed during OMA treatment was at skin level and was observed since the 1st injection. It could be interesting to modify dosage and administration frequency of this biological drug in partially or not responders UV patients.

## Data availability statement

The original contributions presented in the study are included in the article/supplementary material, further inquiries can be directed to the corresponding author.

## Ethics statement

Ethical review and approval was not required for the study on human participants in accordance with the local legislation and institutional requirements. The patients/participants provided their written informed consent to participate in this study.

## Author contributions

FP, DG, SB, ID, VR, PM, and IP made substantial contributions to conception and design, acquisition of data, interpretation of data, reviewed it critically for important intellectual content, given final approval of the version to be published, and agree to be accountable for all aspects of the work related to its accuracy or integrity. FP, DG, and IP drafted the article.

## Conflict of interest

The authors declare that the research was conducted in the absence of any commercial or financial relationships that could be construed as a potential conflict of interest.

## Publisher's note

All claims expressed in this article are solely those of the authors and do not necessarily represent those of their affiliated organizations, or those of the publisher, the editors and the reviewers. Any product that may be evaluated in this article, or claim that may be made by its manufacturer, is not guaranteed or endorsed by the publisher.
